# Potential therapeutic application of gold nanoparticles in B-chronic lymphocytic leukemia (BCLL): enhancing apoptosis

**DOI:** 10.1186/1477-3155-5-4

**Published:** 2007-05-08

**Authors:** Priyabrata Mukherjee, Resham Bhattacharya, Nancy Bone, Yean K Lee, Chitta Ranjan Patra, Shanfeng Wang, Lichun Lu, Charla Secreto, Pataki C Banerjee, Michael J Yaszemski, Neil E Kay, Debabrata Mukhopadhyay

**Affiliations:** 1 Department of Biochemistry and Molecular Biology, 200 1st Street, Mayo Clinic Rochester, MN-55905, USA; 2 Department of Medicine, Division of Hematology, 200 1st Street, Mayo Clinic Rochester, MN-55905, USA; 3 Department of Orthopedic Research, 200 1st Street, Mayo Clinic Rochester, MN-55905, USA; 4 Department of Biomedical Engineering, 200 1st Street, Mayo Clinic Rochester, MN-55905, USA

## Abstract

B-Chronic Lymphocytic Leukemia (CLL) is an incurable disease predominantly characterized by apoptosis resistance. We have previously described a VEGF signaling pathway that generates apoptosis resistance in CLL B cells. We found induction of significantly more apoptosis in CLL B cells by co-culture with an anti-VEGF antibody. To increase the efficacy of these agents in CLL therapy we have focused on the use of gold nanoparticles (GNP). Gold nanoparticles were chosen based on their biocompatibility, very high surface area, ease of characterization and surface functionalization. We attached VEGF antibody (AbVF) to the gold nanoparticles and determined their ability to kill CLL B cells. Gold nanoparticles and their nanoconjugates were characterized using UV-Visible spectroscopy (UV-Vis), transmission electron microscopy (TEM), thermogravimetric analysis (TGA) and X-ray photoelectron spectroscopy (XPS). All the patient samples studied (N = 7) responded to the gold-AbVF treatment with a dose dependent apoptosis of CLL B cells. The induction of apoptosis with gold-AbVF was significantly higher than the CLL cells exposed to only AbVF or GNP. The gold-AbVF treated cells showed significant down regulation of anti-apoptotic proteins and exhibited PARP cleavage. Gold-AbVF treated and GNP treated cells showed internalization of the nanoparticles in early and late endosomes and in multivesicular bodies. Non-coated gold nanoparticles alone were able to induce some levels of apoptosis in CLL B cells. This paper opens up new opportunities in the treatment of CLL-B using gold nanoparticles and integrates nanoscience with therapy in CLL. In future, potential opportunities exist to harness the optoelectronic properties of gold nanoparticles in the treatment of CLL.

## Background

There is increasing evidence that angiogenesis plays a critical role in the pathogenesis of human malignancies [1,2]. Angiogenesis is an event that relies on the formation of vessels from preexisting vasculature that occurs in health and disease. Initially it was found that without new capillary formation there could not be significant tumor growth or metastasis to other organ sites. While the original evidence for this was based on the finding of tissue neovascularization, there have been significant advances delineating the presence of autocrine and/or paracrine pathways in both solid tumors and human leukemias [3,4]. Hematological diseases with aberrant vascularization include; multiple myeloma, acute myeloid leukemia and more recently B-chronic lymphocytic leukemia (CLL). These findings have led to the exciting possibility that strategies that undermine the angiogenic pathways could be used as non-overlapping methods of treatment for these diseases [5].

Initially the secretion of VEGF from malignant tumors was believed to be of primary importance in the development of neovascularization of the tumor involved tissue sites. This important biologic event was associated with more aggressive disease status. However more recently the paracrine role of VEGF has been modified to include autocrine pathway(s) that increase survival of malignant cells in both mouse and human tumor types [6]. Interruption/blockade of the VEGF pathway in those tumor cells has been shown to lead to cell death. To a great extent the level of interruption/blockade has been either to bind VEGF or to inhibit VEGFR-1 or VEGFR-2 [7,8]. Importantly, ourselves and others have found that CLL B cells secrete VEGF and express the VEGF receptors; VEGFR-1, VEGFR-2 and Neuropilin-1 (NRP-1) [9]. The VEGF based pathway appears to be important in the apoptosis resistance of CLL B cells. Thus we have found that culturing CLL B cells with receptor tyrosine kinase inhibitors or anti-VEGF antibodies (Avastin; bevacizumab) leads to increased levels of apoptosis. However, significantly high amount of the antibody was required to have a moderate effect in the apoptosis. In order to enhance the efficacy of agents such as anti-VEGF antibodies we have conducted initial studies utilizing delivery of these antibodies via conjugated gold nanoparticles. The primary rationale for selecting gold nanoparticles is their biocompatibility, very high surface area (large amount of drugs can be loaded), ease of characterization and surface modification (i.e. organic molecules such as drugs, peptides, antibodies, etc. can be easily attached to gold nanoparticles)[10]. This report details our initial work with anti-VEGF (AbVF) conjugated to gold nanoparticles in comparison to naked anti-VEGF antibody or gold nanoparticles alone in the modulation of the apoptotic status of CLL B cells.

## Results and discussion

### Synthesis of gold nanoparticles and conjugation with anti-VEGF antibody

**Figure 1:**
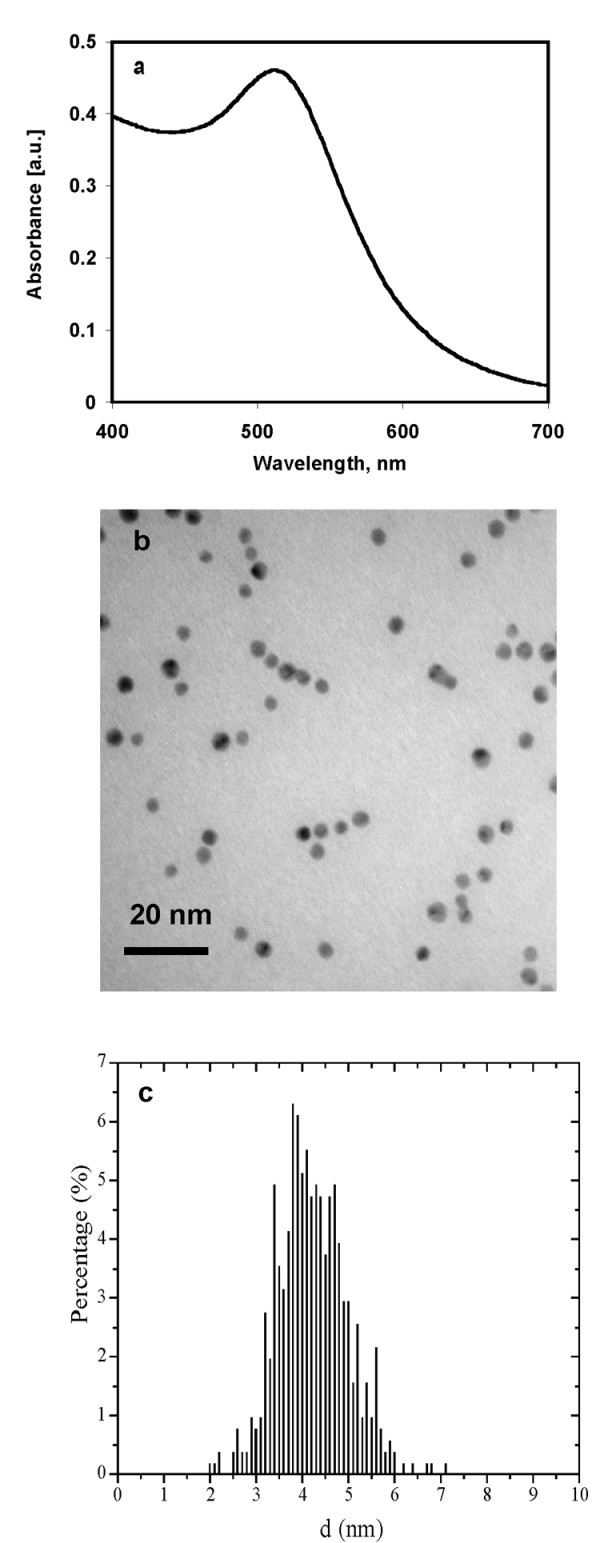
UV-Visible spectroscopy and transmission electron micrograph of gold nanoparticles. a) UV-visible spectrum of a gold nanoparticles solution and b) Transmission electron microscopy picture of gold nanoparticles. TEM was done after drop coating the gold nanoparticles on carbon coated copper grid, c) histogram showing the size distribution of gold nanoparticles.

Gold nanoparticles were synthesized according to standard wet chemical methods using sodium borohydride as a reducing agent [11-13]. Characteristic surface plasmon resonance (SPR) band of gold nanoparticles was observed in the UV-Visible spectrum, confirming the presence of spherical gold nanoparticles (Figure 1a). TEM micrographs showed spherical gold nanoparticles of approximately 4 nm were formed by this method (Figure 1b). Size distribution analysis clearly showed that nearly 90% of the particles reside within 4 nm size range (Figure 1c). Gold nanoparticles obtained by this method were centrifuged at 13,000 rpm for 45 min at 10°C. The loose pellet at the bottom was collected and analyzed for gold content using inductively coupled plasma (ICP) analysis. The concentration of gold was found to be 200 μg/ml. The gold nanoparticles obtained after ultracentrifugation was filtered through 0.22 μM filter paper and UV-irradiated for 15 minutes before their use in the apoptosis assay as control for Au-AbVF.

**Figure 2:**
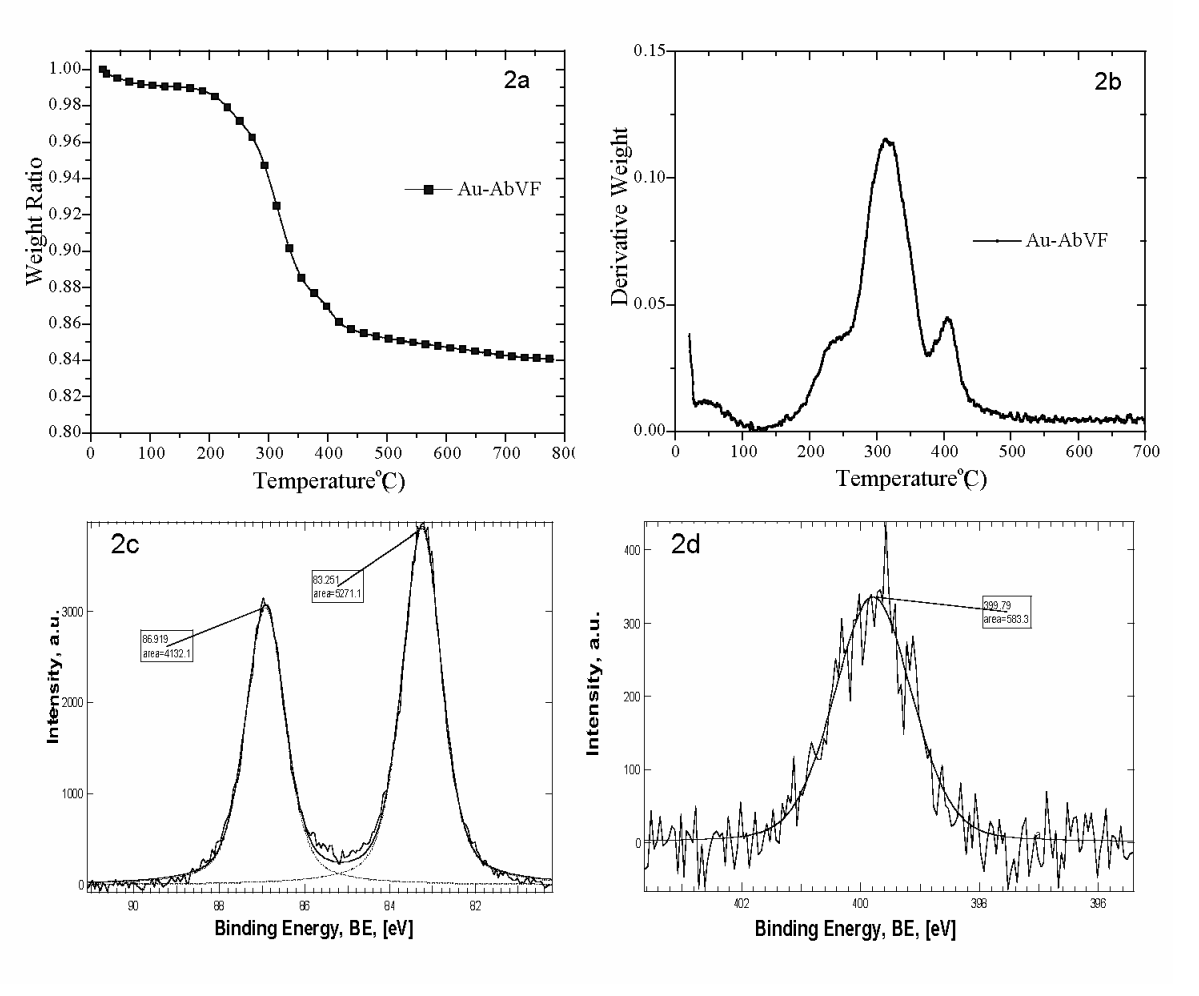
Thermogravimetric analysis (TGA) of Au-AbVF conjugates. For TGA, 150 ml gold nanoparticles were incubated with 600 μg of AbVF. After 1 h, the nanoconjugates were centrifuged at 25000 rpm for 1 h, freeze-dried overnight and analyzed using TGA; 2a) describes weight loss over temperature and 2b) derivative weight loss over temperature for the nanoconjugates. TGA analysis was done on purified and lyophilized nanoconjugates, 2c) X-ray photoelectron spectra of Au-AbVF conjugates. Core level BE of Au and 2d) core level BE of N.

The decision to establish feasibility of anti-VEGF antibody conjugation to gold nanoparticles was based on our earlier observations that anti-VEGF antibodies alone could induce apoptosis of CLL B cells [14]. However we wished to develop maneuvers that would enhance the ability of the anti-VEGF antibody to kill CLL B cells. Attachment of VEGF antibody (AbVF) to gold nanoparticles was done according to published literature and monitored using UV-Visible spectroscopy (Shimadzu, UV2401 PC) because the SPR is very sensitive to surface modification of the gold nanoparticles. An increase in absorbance of gold nanoparticles with a concomitant red shift in the λ_max _was also observed as reported earlier [13]. The increase in absorbance and red shift in the λ_max _indicates the perturbation of the electrical double layer present around the gold nanoparticles on the addition of AbVF and confirms its attachment on the gold nanoparticles [13]. Finally, the concentration of AbVF on gold nanoconjugates and its nature of bonding with gold nanoparticles were determined using thermogravimetric analysis (TGA) and X-ray photoelectron spectroscopy (XPS). For TGA, 150 ml gold nanoparticles were incubated with 600 μg of AbVF. After 1 h, the nanoconjugates were centrifuged at 25000 rpm for 1 h, freeze-dried overnight and analyzed using TGA. Figure 2a and 2b describe the TGA profile of Au-AbVF nanoconjugates. Figure 2a clearly shows a total weight loss of 12% spanning over three distinct steps of weight losses at 200°C, 250°C and 375°C. The graph of derivative weight loss over temperature (Fig. 2b) shows three distinct maxima of weight losses at nearly 240°C, 320°C and 400°C. A weight loss of 13.3% should be observed according to theoretical calculation (where 1 ml gold nanoparticles solution containing 26 μg of gold was incubated with 4 μg of AbVF). Therefore, a total weight loss of 12% accounts for 90% of the AbVF used initially for attachment was actually bound to gold nanoparticles.

Three different weight losses at three different temperatures indicate that there are, at least, three different modes of bonding between AbVF and gold nanoparticles. Gold is known to bind strongly with organic molecules containing thiols and amines groups [18]. The formation of self-assembled monolayers of organothiols on gold surfaces has been attributed to its ability to bind thiolates covalently [16,17]. Since the discovery of immunogold labeling in 1971 by Faulk and Taylor, a great deal of information is known about the nature of bonding between gold nanoparticles and antibodies [18]. It is now well recognized that there are, at least, three separate but dependent phenomenon that might explain the bonding of AbVF to gold nanoparticles; i) electrostatic attraction of negatively charged gold nanoparticles with positively charged protein molecules, ii) covalent bonding between the thiols/amine groups present within the amino acids in the antibody and the gold nanoparticles, and iii) hydrophobic interaction between proteins and gold nanoparticles [19]. Our data suggests that after being electrostatically attracted to gold nanoparticles, AbVF (having a lysine and cysteine residues) binds covalently to the gold nanoparticles through the thiol/amine groups. The small amount of weight losses at relatively lower temperature is suggestive of a weaker interaction between gold and the antibody and we speculate it to be hydrophobic in nature. A second weight loss at higher temperature (320°C) indicates another distinctly different bonding mode that needed much higher energy to desorb/decompose the antibody from the nano-gold surfaces. The maximum weight loss at this temperature is most likely due to the presence of gold-sulfhydryl/gold-amine bond where the antibody is held to the nano gold surface through covalent interaction between the gold nanoparticles and cysteine/lysine residues present in the antibody [20]. A third weight loss at the even higher temperature (400°C) was suggestive of the presence of a third complex mode of attachment of the antibody to gold nanoparticles that requires higher energy to desorb/decompose from the nanogold surfaces.

The nature of bonding between gold and AbVF was further supported by X-ray photoelectron spectroscopy (XPS). A single gold (Au 4f7/2) peak at 83.2 eV with a spin orbit coupling of 3.7 eV was observed in the drop-coated conjugates. These data clearly demonstrate that all of the Au^+3 ^ions used in the process were reduced to Au^0 ^by sodium borohydride (Fig. 2c) [11,21]. Two weak sulfur peaks were also observed. The presence of two sulfur peaks at 162.7 and 167.1 eV represents two chemically distinct sulfur species (data not shown). We observed similar sulphur peaks from gold conjugates containing VEGF165, a growth factor [11]. This is not unexpected because both the VEGF165 and AbVF are proteins and it is reasonable to assume that they use similar chemical entities such as cysteine or lysine residues to bind to gold nanoparticles. The peak at 162.7 eV can be assigned to gold-thiolate bond and peaks at higher binding energy (BE) to sulfones, an oxidized sulfur species. The origin of this sulfone peak may arise due to aerial oxidation of the sulfur during sample preparation [11]. Unbound thiol peaks normally appear at 164 eV [21]. The nitrogen 1s peak at 399.6 eV (Fig 2d) is likely due to unionized, non-protonated nitrogen [22]. This is in agreement with earlier studies reported on the adsorption of proteins/amino acids or amines on gold surfaces [22,23]. Therefore, we speculate that AbVF may bind to gold nanoparticles through -NH_2 _functionalities via pseudo-covalent interaction. Hence, we infer from the XPS and TGA studies that AbVF binds to gold nanoparticles through sulfur and/or nitrogen present in the cysteine/lysine residues in the antibody. Following the establishment of a protocol that we could reliably generate and characterize gold nanoparticles conjugated to AbVF we then explored the ability of this conjugate to alter leukemic B cell apoptosis. For the apoptosis experiments, 150 ml gold nanoparticles were incubated with 600 μg of AbVF and purified through ultracentrifugation as described above and then used for the study after UV-Irradiating the particles for 20–30 minutes. Exactly the same amount of gold nanoparticles (Au) and AbVF present in the respective doses of Au-AbVF were separately used as controls. It is important to mention here that in the case of AbVF and Au-AbVF treated groups, the doses are always referred to based on the amount of AbVF present in the nanoconjugates, e. g, 25 μg Au-AbVF corresponds to 25 μg AbVF present in the nanoconjugates. However, in the case of gold treated groups the doses of 1, 5 and 25 μg correspond to the exactly same amount of gold present in 1, 5, and 25 μg of Au-AbVF respectively.

### Impact of gold nanoparticles (Au), and gold nanoparticles conjugated with AbVF (Au-AbVF) and AbVF on CLL B cell apoptosis

**Figure 3:**
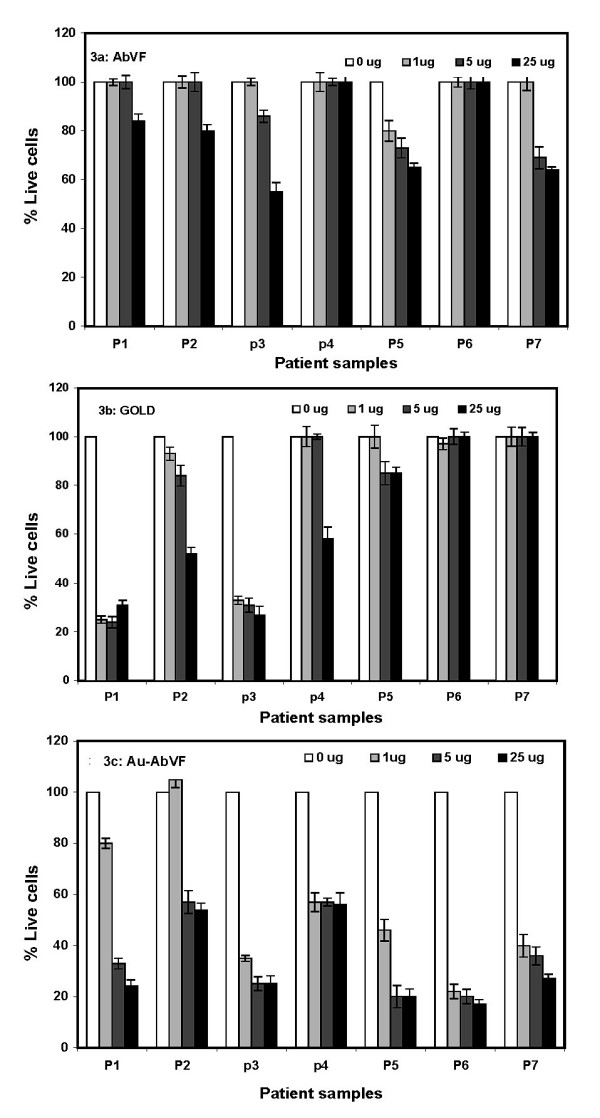
Dose dependent effect of AbVF, Au and Au-AbVF in the induction of apoptosis in CLL-B cells. Cells were treated with 1, 5 and 25 ug of AbVF/Au/Au-AbVF for 72 h followed by measurement of apoptosis using annexin PI. The % apoptosis was calculated after normalizing the apoptosis in the control cells (no treatment group) to zero; 3a) effect of AbVF to induce apoptosis in CLL-B cells; 3b) effect of Au nanoparticles only to induce apoptosis in CLL-B and figure 3c) effect of Au-AbVF to induce apoptosis in CLL B cells. In the case of AbVF treated group, the amount of AbVF used is exactly the same in the corresponding doses of Au-AbVF. Similarly, in case of gold treated group, the amount of gold nanoparticles used is exactly the same as in the corresponding doses of Au-AbVF.

It is also important to note here that there is hardly any preclinical model available for CLL-B studies. Therefore, studies with primary CLL-B cells (cells isolated directly from patient blood) are considered as a preclinical study. Figure 3 describes the dose dependent behavior of AbVF, Au and Au-AbVF in inducing apoptosis of CLL B cells isolated from 7 different patients. CLL B cells were incubated with AbVF, Au and Au-AbVF separately for 72 hours followed by apoptosis measurement using Annexin/PI analysis. Figure 3a describes the effect of different doses of AbVF in inducing apoptosis.

Among the 7 samples, 3 samples (P3, P5 and P7) showed moderate apoptosis levels (~40%) in a dose dependent manner; maximum apoptosis was observed at highest dose (25 μg). For the remaining 4 samples, 2 showed no effect (Sample P4 and P6) and 2 showed only a partial apoptosis above baseline (~10%). Figure 3b describes the effect of gold nanoparticles alone in the induction of apoptosis in CLL-B cells. The amount of gold nanoparticles used is exactly the same as in the respective doses of Au-AbVF. From this figure it is clear that 3 (P1, P2, P3) samples responded to the exposure to gold nanoparticles alone with increases in apoptosis (~50–60% apoptosis was observed) in a dose dependent manner. Among the remaining 4 samples, 2 did not respond at all (P6, P7) and lesser levels of apoptosis induction were observed for the remaining 2 samples (P4, P5). Figure 3c describes the effect of Au-AbVF in the induction of apoptosis in CLL-B cells isolated from patients. From figure 3c it is evident that all the 7 samples responded more effectively to the gold-AbVF treatments in terms of apoptosis induction. Significant increases in apoptosis (~80%) in a dose dependent manner were observed in 4 out of the 7 CLL samples and a significant enhancement in the apoptosis was observed in all the samples compared to Au and AbVF controls. The apoptosis induction observed in the case of control gold nanoparticles (Au treatment group) on CLL B cells is not surprising as we have shown previously that the gold nanoparticles posses unique antiangiogenic properties [11-13]. Recently, it has been reported that gold nanoparticles inhibit the function of heparin binding growth factors such as VEGF165, BFGF and posses anti-angiogenic properties. Therefore, gold nanoparticles alone without any modification can inhibit the function of some of the growth factors secreted by the CLL B cells and hence the induction of apoptosis [11,12].

**Figure 4:**
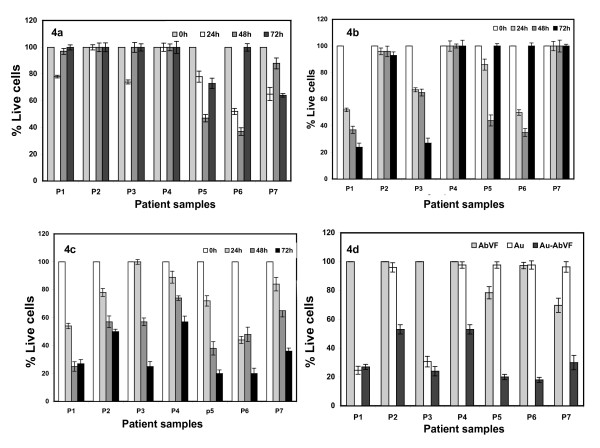
Effect of time on the induction of apoptosis of CLL-B cells by AbVF, Au and Au-AbVF. Cells were treated with 5 ug/ml of AbVF/Au/Au-AbVF for 72 h. Apoptosis measurement was then done using annexin/PI and apoptosis was calculated as described above; 4a) effect of AbVF alone; 4b) effect of Au alone; 4c) effect of Au-AbVF alone and 3d) comparison of the efficacy of AbVF, gold and gold-AbVF, 4d) comparison of effect of AbVF, Au and Au-AbVF on the apoptosis of CLL-B cells. To compare the activity of AbVF either conjugated or free the amount of AbVF used as control is exactly the same amount of AbVF present in the nanoconjugate. Similarly, to compare the activity of Au and Au-AbVF the amount of Au used as control is exactly the same amount of Au present in the nanoconjugate. The quantitations of Au in the nanoparticles solution and in the purified nanoconjugates were determined using inductively couple plasma analysis (ICP).

Figure 4 describes the time course of the effect of AbVF, Au and Au-AbVF in the induction of apoptosis to CLL-B cells. CLL-B cells were treated with 5 μg/ml of AbVF either bound or non-bound to gold as well as gold controls separately for 24 h, 48 h and 72 h respectively (the amount of gold in the control Au-treated good is exactly the same here as in 5 μg of Au-AbVF). Figure 4a shows the effect of non-conjugated AbVF to induce apoptosis in CLL-B cells treated with 5 μg/ml of AbVF. After the respective time points, cells were analyzed for apoptosis using annexin/PI method. Figure 4a shows the effect of non-conjugated AbVF over time and a clear level of apoptosis induction over baseline was observed in 3 samples out of 7 (P5, P6, P7). However, no clear time dependent apoptosis was observed with the 5 μg/ml dose. Figure 4b describes the effect of Au nanoparticles on the time dependent induction of apoptosis of CLL-B cells (n = 7). Time dependent apoptosis was observed for P1 and P3 where maximum apoptosis was observed at 72 h, however P2, P4 and P7 did not respond with apoptosis induction to the nanogold particles. For P5 and P6, time dependent apoptosis was observed for first 48 h, however, no apoptosis was observed at 72 h. We believe these residual B cells were resistant to the nonconjugated Au nanoparticles. The degree of apoptosis observed from the treatment with gold nanoparticles alone is not unexpected due to the anti-angiogenic properties of gold nanoparticles, as described above. Figure 4c describes the effect of Au-AbVF in the induction of apoptosis in CLL B cells. With this conjugate time dependent apoptosis was observed with maximum apoptosis observed at 72 h for all CLL B cells. Importantly in the case of Au-AbVF treated samples for both experiments depicted in figure 3 and 4, all the CLL B cell samples showed significantly higher induction in apoptosis (2–5 fold) compared to either the AbVF treatment group or gold treatment groups (Fig 4d). It is also important to mention here that control experiments with same doses of nanoconjugates used in CLL-B cell studies using peripheral blood mononuclear cells (PBMCs) isolated from normal healthy individual did not induce significant apoptosis. Only a less than 10% apoptosis was observed with the highest does of Au alone or AbVF or Au-AbVF whereas the lower doses did not induce any significant apoptosis.

**Figure 5:**
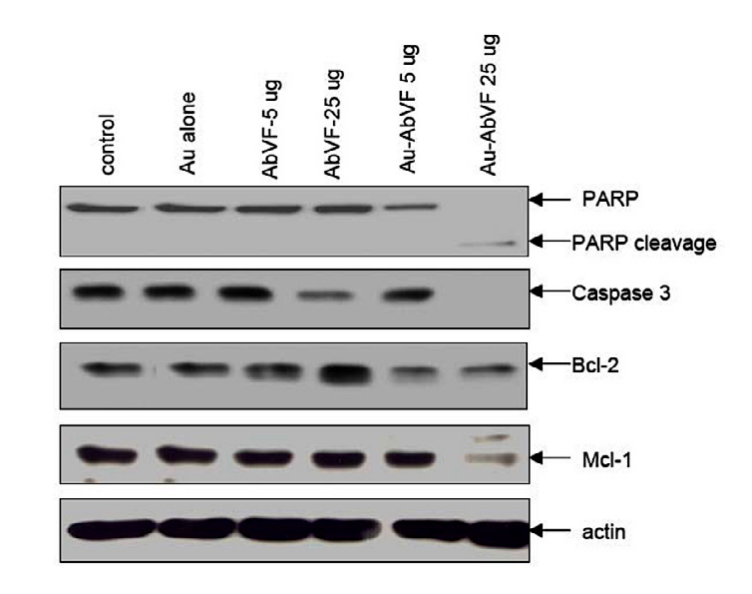
Immunoblot analysis of CLL B cells exposed to gold particles (Au) alone, AbVF alone at 5 and 25 μg/ml and AU-AbVF at 5 and 25 μg/ml. Note the prominent PARP cleavage, decrease in caspase 3, Mcl-1 and moderate change in Bcl-2 for CLL B cells treated with Au-AbVF at 25 μg/ml. These changes were noted but less evident for Au-AbVF at the 5 μg/ml dose.

Various autocrine pathways in CLL B cells provide important survival advantages for these cells. We have already established that a VEGF autocrine pathway exists in CLL B cells [7]. The evidence for this includes our finding that recombinant VEGF165 can rescue CLL B cells from both spontaneous and drug-induced apoptosis. The addition of VEGF to CLL B cells also resulted in significant increases in the antiapoptotic proteins Mcl-1 and XIAP. In addition we found that the two VEGF receptors (VEGF-R1 and VEGF-R2) on CLL B cells are spontaneously phosphorylated. The combination of VEGF-induced increases in anti-apoptotic proteins combined with the finding of phosphorylated VEGF receptors strongly suggested that a VEGF-based pathway is linked to CLL Bcell survival. We consistently found that the anti-apoptotic protein Mcl-1 was increased when CLL B cells were exposed to exogenous VEGF165 [3]. To find out the mechanism of more effective induction in apoptosis in CLL-B cells by Au-AbVF conjugates, we looked at the levels of Mcl-1, PARP, BcL-2 and caspase3 in CLL-B cells treated with different doses of Au, AbVF and Au-AbVF. Figure 5, shows that CLL B cells have clearly detectable PARP cleavage, decrease in caspase-3, Mcl-1 and Bcl-2 for Au-AbVF exposed cells but not for Au control or AbVF alone. Thus we believe that the AbVF conjugated to gold nanoparticles are inducing apoptosis in the CLL B cells similar to what we have found with VEGF pathway blockade using bevacizumab alone, but Au-AbVF is more effective than naked antibody 2C3 (i.e. 2–5 fold increase in induction of apoptosis). Previously, we have shown our ability to bind anti-angiogenic molecules and anti-cancer drugs on a single gold core in a "spoke in a wheel" fashion [13]. This study details our ability to enhance the efficacy of anti-VEGF antibody, 2C3, when conjugated to gold nanoparticles with resultant significant enhancement in apoptosis of primary CLL B cells when compared to an anti-VEGF antibody or gold nanoparticles alone. The reason for such an enhanced activity of Au-AbVF towards apoptosis induction is currently unknown and a subject of future investigation, but we believe that enhancing efficacy of the antibodies and lowering of the doses when delivered as gold nanoconjugates will have tremendous implications in the treatment of CLL.

### Fate of gold nanoparticles and its conjugates after treatment

**Figure 6:**
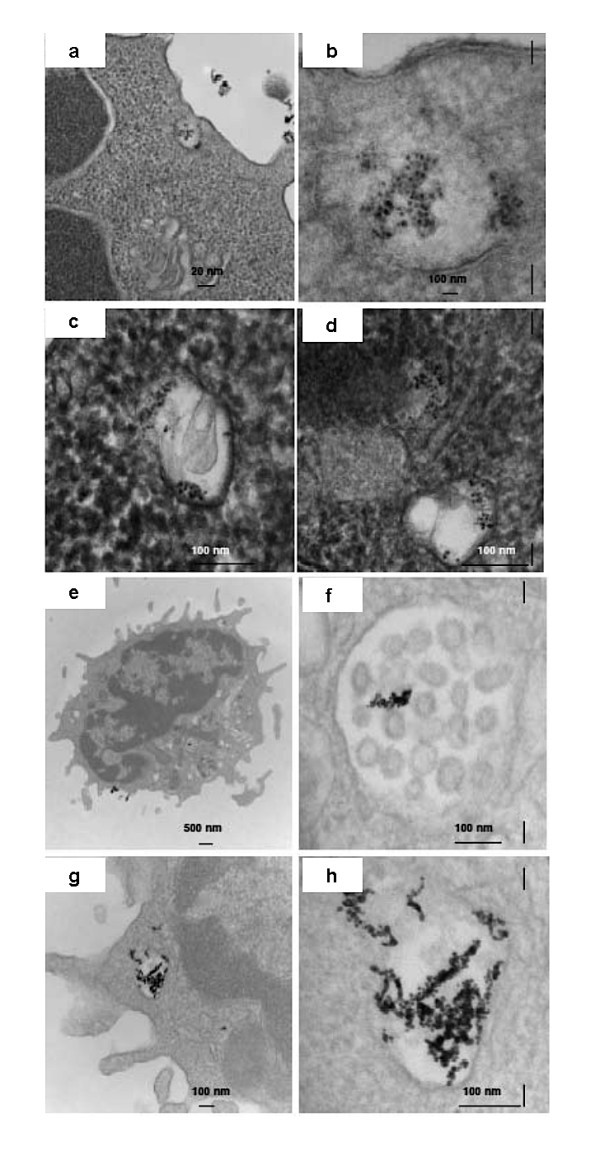
Internalization of Au-AbVF and Au alone by primary CLL-B cells after 1 h incubation. 6a) nanoparticles were seen at the cell periphery (within uncoated tubules and vacuoles); 6b) higher magnification image of 6a; 6c and 6d) showing the internalized particles in different endocytic compartments. Figure 6e to 6h show the internalization of Au nanoparticles alone by primary CLL-B cells after 1 h incubation. 6e) Nanoparticles were seen at the cell periphery (within uncoated tubules and vacuoles); 6f) higher magnification image of 6e; 6f) showing the internalized particles in multivesicular bodies; and 6g) higher magnification image of 6f.

It is also important to know the fate of the nanoparticles after the treatment. Do the cells internalize the particles or do the particles remain bound to the cell membrane? To address these issues transmission electron microscopy of the cells treated with gold nanoparticles alone and with Au-AbVF for 1 h is performed [24]. The 1 h treatment duration was chosen based on previously reported literature on the internalization of gold nanoparticles as longer time points did not increase gold uptake nor does it alter the pattern of internalization. Figure 6 exhibits the internalization of Au-AbVF by primary CLL-B cells after 1 h treatment. All the features of early as well as late internalization were clearly visible. Nanoconjugates were found at the cell periphery (within uncoated tubules and vacuoles, Figure 6a, b). Gold nanoparticles were also detected within larger endocytic compartments of diverse morphology. These include peripherally both early and late endosomes and lysozomes (6c, 6d). Similar pattern of internalization was observed when the cells were treated with gold nanoparticles alone (Figure 6e–6h). However, in this case, the number of gold nanoparticles taken up by the cells was found to be much lower than the previous case and lots of aggregated gold nanoparticles were seen. Since, the focus of this paper is not to find out the endocytic pathway of gold nanoparticles/gold nanoconjugates by primary CLL-cells, so further detailed studies to find out the mechanisms of internalization was not performed. But, we speculate that the particles may be taken up by classical pinocytic mechanism of engulfing particles of very smaller size.

While we may be concerned about the possible toxicities of gold nanoparticles in human therapies, colloidal gold actually has a long history of use in humans [25,26]. The therapeutic use of gold can be traced back to the Chinese in 2500 BC [10]. They were the first to prepare and use red colloidal gold as the "drug of longevity." Red colloidal gold is still in use today in India in the form of Ayurvedic medicine for rejuvenation and revitalization during old age under the name of Swarna Bhasma ("Swarna" meaning gold, "Bhasma" meaning ash) [27,28]. Further evidence of gold nanoparticles as potential non-toxic agents comes from our murine preclinical model in which we show that intraperitoneal administration of gold nanoparticles to C3H mice does not cause any acute biochemical and/or hematological toxicities [13]. *In vitro*, the non-toxic behavior of gold nanoparticles has also been addressed by other groups including ours [29-31]. Another recent report has shown in a preclinical mouse model, that doses of gold nanoparticles as high as 2.7 g/kg body weights did not cause any significant toxicity in mice up to 1 year of study [32]. Furthermore, light absorbing properties of gold nanoparticles has been exploited to inflict localized damage to cells at sublethal levels to transiently modify cellular functions [33]. Gold-silica nanoshell has been used in nanoshellassisted photo-thermal therapy (NAPT) to kill tumor cells by near infrared light (NIR) [34]. Gold-EGFR conjugates have been used to detect the cancer cells by exploiting the light scattering properties of gold nanoconjugates [35]. This paper is unique in that it bridges the field of nanoscience and technology with the therapy in CLL and opens up the opportunity of utilizing the technological advances of nanoscience in the treatment of CLL.

These findings in CLL reinforce the advantages of using gold-nanoparticle-based drug delivery system in human malignancies. They include; increased concentration of drug loading due to high surface area of the nanoparticles, the ability to load multiple drugs (including the targeting agent) on the nanoparticle and the important feature of achieving higher local drug concentrations with reduced systemic toxicity and enhanced efficacy. We continue to develop this nanoparticle system in order to not only exploit the use of gold nanoparticles as anti-angiogenic targeted molecules but to conjugate other known chemotherapeutic (i.e. fludarabine) or monoclonal agents (i.e. rituximab) to these nanoparticles as well. This delivery system has the potential to induce cell death in CLL B cells by not only interrupting the VEGF based survival pathway but also by delivering effective levels of chemoimmunotherapeutics to the cells. In future, potential opportunities exist to harness the size dependent optoelectronic properties of gold nanoparticles in the treatment of CLL.

## Materials and methods

### Patient selection and purification of lymphocytes

Blood was obtained from healthy donors or CLL patients who had provided written informed consent. The laboratory study was approved by the Mayo Clinic Institutional Review Board according to the regulations of the Declaration of Helsinki. All CLL patients had a confirmed diagnosis using the NCI working group definition [36]. Patients in this cohort were from all Rai stages and had not been treated for at least 6 weeks prior to blood processing for this study. Peripheral blood mononuclear cells (PBMC) were separated from heparinized venous blood by density gradient centrifugation. To remove adherent cells, PBMC were suspended in RPMI 1640 supplemented with 10% fetal calf serum (FCS) and incubated in plastic dishes at 37.1°C for 1 h prior to collection of non-adherent cells. To obtain at least 95% purity of CLL B cells, non-adherent cells were depleted of T cells by incubation with sheep erythrocytes. In addition, we also purified CLL B cells from magnetic bead columns. To do this, highly purified CD19^+ ^B cells (≥95%) were obtained from PBMC by standard negative selection using a cocktail of subset-specific antibodies conjugated with magnetic beads (Miltenyi Biotech, Auburn, CA, USA). These purified CLL B cells were then either used immediately for the laboratory studies described below or cryopreserved in RPMI 1640, 20% FCS, and 10% DMSO and stored in liquid nitrogen until use.

### Cell culture

Primary CLL B cells and human splenic B cells obtained from controls were sorted by CD 19 antibody conjugated to magnetic beads (Miltenyi Biotec, Auburn, CA, USA) and were then cultured in serumfree AIM-V (Gibco BRL, USA) and RPMI (Biomol, USA) supplemented with 10% FCS, respectively. Cells were maintained at 37°C in an atmosphere containing 95% air-5% CO_2 _(v/v).

### Reagents

Immunological reagents that recognize the following antigens were purchased from the indicated suppliers: Mcl-1 from BD Pharmingen (San Diego, CA, USA); Bcl-2 from Dako Corp (Carpinteria, CA, USA); XIAP from R&D Systems, Inc. (Minneapolis, MN, USA); β-actin from Novus Biologicals (Littleton, CO, USA). VEGF neutralizing antibody (2C3) was a kind gift from Pergerine Pharmaceuticals. The antibody (2C3) specifically blocks the interaction between VEGF and VEGFR-2 [37].

### Synthesis and characterization of gold nanoparticles and its nanoconjugates

Gold nanoparticles were synthesized according to standard wet chemical methods using sodium borohydride as a reducing agent [11-13]. Characteristic surface plasmon resonance (SPR) band of gold nanoparticles was observed in the UV-Visible spectrum, confirming the presence of spherical gold nanoparticles (Fig. 1a). TEM micrographs showed spherical gold nanoparticles of approximately 5 nm were formed by this method (Fig. 1b). The size distribution analysis of gold nanoparticles (after counting 500 individual particles) clearly shows that most of the gold nanoparticles (~90%) are in 4–5 nm range (Fig 1c).

Binding of VEGF antibody to gold nanoparticles was done according to our previously published report [13]. In brief, 150 ml of gold nanoparticles were incubated with 600 μg of AbVF for 1 h at room temperature under stirring. After 1 h, gold conjugates thus obtained were ultracentrifuged at 25000 rpm at 10°C for 1 h. The loose pellet obtained at the bottom was collected and UV-irradiated for 20–30 minutes before use in apoptosis assays. The binding was monitored using UV-Visible spectroscopy. UV-Visible spectrum was recorded on a Shimadzu model system (UV2401 PC) and the saturation concentration was determined.

### Quantitation of AbVF in the nanoconjugates using thermogravimetric analysis (TGA)

For TGA analysis, 150 ml of gold nanoparticles were incubated with 600 μg of AbVF for 1 h at room temperature under stirring. After 1 h, gold conjugates were centrifuged at 25000 rpm at 10°C for 1 h. Both the supernatant and the loose pellet at the bottom were collected. The loose pellet obtained after ultracentrifugation was used for the quantification of AbVF attached on gold nanoparticles and also for apoptosis studies with CLL B cells described below. For TGA analysis, the pellet obtained after ultracentrifugation was lyophilized to obtain gold nanoconjugates in powder form. The lyophilized powder of nanoconjugates was used for TGA analysis. TGA was done using a TA Instruments Q500 thermal analyst. The TGA data were obtained in flowing nitrogen at a heating rate of 20°C/min. Amount of drugs attached onto gold nanoparticles were obtained from weight loss from the TGA curve as previously described [10].

### Studying nature of bonding by X-ray photoelectron spectroscopy

X-ray photoelectron spectroscopy (XPS) was performed to find out the nature of bonding between gold nanoparticles and the antibody. XPS was obtained on a PHI 5400 instrument using a Mg Kα Xray (1253.6 eV) anode source operated at 250 W, pressure was below 2 × 10^-9 ^torr. The electron pass energy on the hemispherical analyzer was set at 89.45 eV for survey scans and 17.9 eV for highresolution scans. The binding energy scale was referenced to that of C1s (285.0 eV). Samples were prepared by drop-coating anti-VEGF165 antibody conjugated gold solution on a clean silicon wafer and the drops were allowed to air dry [38].

### Studying apoptosis in CLL-B cells isolated from patients

To compare gold nanoconjugates containing AbVF (Au-AbVF) to AbVF alone, a humanized anti-VEGF-A monoclonal antibody 2C3 (Pergerine Pharma) was co-cultured with purified CLL B primary cells. In brief, CLL B cells (1 × 10^6^) were co-cultured with increasing concentrations of 2C3 conjugated to gold as well as 2C3 and gold (1 μg–25 μg/ml) alone as controls for 24–72 hours. Annexin/PI flow cytometry was done on those cells according to our prior procedures [3]. We wish to indicate here that all the experiments reported here were repeated in triplicate.

### Immunoblotting analysis

To detect alterations of apoptotic proteins and confirm apoptosis we used immunoblot analysis. Primary CLL B cells were cultured in six-well tissue culture dishes and treated with either vehicle (control) or Au alone, AbVF, Au-AbVF for 24 h. The treated cells were then washed three times with calcium, magnesium-free Dulbecco's phosphate-buffered saline (PBS) and solubilized in alkylation buffer (6 M guanidine hydrochloride, 250 mM Tris-HCl, pH 7.4 at 21.1°C, and 10 mM EDTA supplemented immediately before use with 150 mM 2-mecaptoethanol and 1 mM phenylmethylsulfonyl fluoride). Samples were then dialyzed into 4 M urea and then 0.1% SDS. Protein extracts were then separated on 5–15% SDS-PAGE acrylamide gels and transferred to nitrocellulose membrane and blocked with 10% Tris-saline milk (TSM). Relevant primary antibodies were diluted in the 10% TSM milk and incubated overnight on the shaker at room temperature. After three extensive washing with 1 × PBS containing 0.05% Tween 20 for 15 min, the blot was then incubated with horseradish peroxidase-conjugated secondary antibody (KPL Inc., Gaithersburg, MD, USA) diluted 1:8000 in 10% TSM for 1 h at room temperature. After washing with 1× PBS containing 0.05% Tween 20 for another 30 min, bound antibodies were detected by ECL chemiluminescence (Amersham Pharmacia, Piscataway, NJ, USA).

### Transmission electron microscopy

TEM sample preparation involving cells, however, was performed by treating cells with gold nanoparticles and gold nanoconjugates for 1 h with under serum-free conditions. After the incubation, CLL-B cells were centrifuged initially at 2500 rpm for 6 min. The resultant cell pellets were then washed thrice with PBS, and fixed in Trump's fixative (1% glutaraldehyde and 4% formaldehyde in 0.1 M phosphate buffer, pH 7.2). Both cell types were then rinsed for 30 min in 3 changes of 0.1 M phosphate buffer, pH 7.2, followed by a 1 hr postfix in phosphate-buffered 1% OsO_4_. After rinsing in 3 changes of distilled water for 30 min, the tissue was en bloc stained with 2% uranyl acetate for 30 min at 60°C. The cell was then rinsed in three changes of distilled water, dehydrated in progressively higher concentrations of ethanol and 100% propylene oxide, and embedded in Spurr's resin. Thin (90 nm) sections were cut on a Reichert Ultracut E ultramicrotome, placed on 200 mesh copper grids, and stained with lead citrate. Micrographs were taken on a TECNAI 12 operating at 120 KV.
