# Correction: potential therapeutic application of gold nanoparticles in B-chronic lymphocytic leukemia (BCLL): enhancing apoptosis

**DOI:** 10.1186/1477-3155-11-23

**Published:** 2013-07-01

**Authors:** Priyabrata Mukherjee, Resham Bhattacharya, Nancy Bone, Yean K Lee, Chitta Ranjan Patra, Shanfeng Wang, Lichun Lu, Charla Secreto, Pataki C Banerjee, Michael J Yaszemski, Neil E Kay, Debabrata Mukhopadhyay

**Affiliations:** 1 Department of Biochemistry and Molecular Biology, Mayo Clinic Rochester, 200 1st Street, Rochester, MN 55905, USA; 2 Department of Medicine, Division of Hematology, Mayo Clinic Rochester, 200 1st Street, Rochester, MN 55905, USA; 3 Department of Orthopedic Research, Mayo Clinic, 200 1st Street, Rochester, MN 55905, USA; 4 Department of Biomedical Engineering, Mayo Clinic Rochester, 200 1st Street, Rochester, MN 55905, USA; 5 Current address: Department of Chemical Biology, Indian Institute of Chemical Technology [IICT], Tarnaka, Hyderabad-607, Andhra Pradesh, India; 6 Current address: Department of Materials Science and Engineering, and Institute of Biomedical Engineering, The University of Tennessee, Knoxville, TN 37996-2200, USA; 7 Current address: Department of Metallurgical and Material Engineering, Jadavpur University, Kolkata 700032, India

## Correction

After the publication of this work [1], it was brought to our attention that there might have been an error during compilation of the gel bands in Figure 1. We regret such unintentional errors and any inconvenience this may have caused. In order to ensure that the published figure is correct, we have repeated the experiments to provide a corrected version of Figure 1.

**Figure 1:**
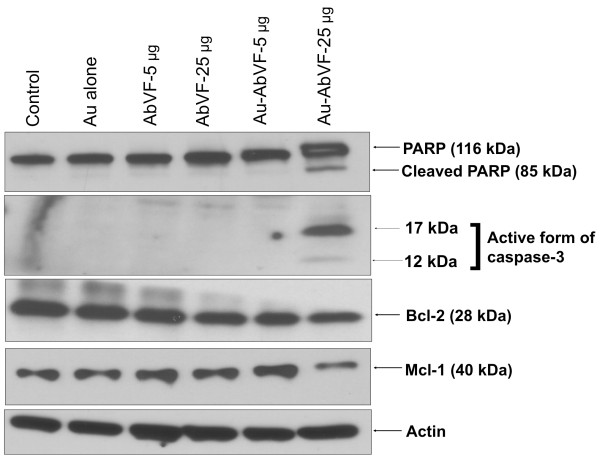
**Immunoblot analysis of CLL B cells exposed to gold particles (Au) alone, AbVF alone at 5 and 25 μg/ml and AU-AbVF at 5 and 25 μg/ml.** Note the prominent PARP cleavage, decrease in caspase 3, Mcl-1 and moderate change in Bcl-2 for CLL B cells treated with Au-AbVF at 25 μg/ml. These changes were noted but less evident for Au-AbVF at the 5 μg/ml dose.
